# Are Metastases from Metastases Clinical Relevant? Computer Modelling of Cancer Spread in a Case of Hepatocellular Carcinoma

**DOI:** 10.1371/journal.pone.0035689

**Published:** 2012-04-23

**Authors:** Anja Bethge, Udo Schumacher, Andreas Wree, Gero Wedemann

**Affiliations:** 1 Competence Center Bioinformatics, Institute for Applied Computer Science, University of Applied Sciences Stralsund, Stralsund, Germany; 2 Institute for Anatomy and Experimental Morphology, University Medical Center Hamburg-Eppendorf, Hamburg, Germany; 3 Institute of Anatomy, University of Rostock, Rostock, Germany; Dana-Farber Cancer Institute, United States of America

## Abstract

**Background:**

Metastasis formation remains an enigmatic process and one of the main questions recently asked is whether metastases are able to generate further metastases. Different models have been proposed to answer this question; however, their clinical significance remains unclear. Therefore a computer model was developed that permits comparison of the different models quantitatively with clinical data and that additionally predicts the outcome of treatment interventions.

**Methods:**

The computer model is based on discrete events simulation approach. On the basis of a case from an untreated patient with hepatocellular carcinoma and its multiple metastases in the liver, it was evaluated whether metastases are able to metastasise and in particular if late disseminated tumour cells are still capable to form metastases. Additionally, the resection of the primary tumour was simulated. The simulation results were compared with clinical data.

**Results:**

The simulation results reveal that the number of metastases varies significantly between scenarios where metastases metastasise and scenarios where they do not. In contrast, the total tumour mass is nearly unaffected by the two different modes of metastasis formation. Furthermore, the results provide evidence that metastasis formation is an early event and that late disseminated tumour cells are still capable of forming metastases. Simulations also allow estimating how the resection of the primary tumour delays the patient's death.

**Conclusion:**

The simulation results indicate that for this particular case of a hepatocellular carcinoma late metastases, i.e., metastases from metastases, are irrelevant in terms of total tumour mass. Hence metastases seeded from metastases are clinically irrelevant in our model system. Only the first metastases seeded from the primary tumour contribute significantly to the tumour burden and thus cause the patient's death.

## Introduction

Metastases are the main cause of death in cancer patients [1]. As a consequence the control of metastases formation has become one of the fundamental goals in cancer treatment. In order to treat metastases formation it is important to understand the processes underlying the metastatic progression. Extensive research had been done in this area within the last decades. Despite substantial insights being gained (e.g. [2]–[6]) many crucial issues are still open, some of which are highlighted in several reviews [7]–[12].

Two major models of metastatic progression have been developed: the linear and the parallel progression model. In the linear progression model it is assumed that the primary tumour undergoes several rounds of genetic alterations and competitive selection before highly malignant cells are able to disseminate and seed metastases into distant organs. Since these cells are already highly malignant, the newly created metastases are also likely able to spawn further new metastases [13]. The parallel progression places the dissemination of tumour cells early in the development of the primary tumour, when the cells have not gained full malignant potential. During outgrowth to a macroscopic detectable metastasis they adapt to the distant sites, which leads to genetic disparity between primary tumour cells and cells within metastases. It is even assumed that signals from the primary tumour might promote the outgrowth from the metastatic site. Late disseminated tumour cells might be less capable to form metastases or might not even be relevant for the patient's death [13].

Although differing in even fundamental aspects of metastasis formation, both of those models raise four fundamental questions: 1) When does the dissemination of malignant cells from the primary tumour starts? 2) Are metastases able to metastasize? 3) Are late disseminated tumour cells capable to form metastases? 4) Does the primary tumour support the outgrowth of metastases via signals?

To answer these four questions, mathematical models are highly versatile tools in doing so as their predictions can be examined and verified quantitatively. A great variety of mathematical models have been developed, ranging from very fine-grained ones that consider very detailed biological and molecular or even genetic characteristics of individual tumour cells [14] to more descriptive models that concentrate on the whole process, like number of cells, size or number of metastases [15]–[17]. All these so far published models only describe a specific aspect of malignant progression, e. g. to model the untreated course [15], to estimate the survival time [16], to predict drug pharmacokinetics [18], [19] or to estimate the effective radiation dose [20].

We therefore wanted to create a computer model which is able to describe the entire aspects of the metastatic cascade. It allows us to model different metastatic characteristics, such as the ability of metastases to metastasize or a variation of the starting point of metastasis. It can furthermore simulate the effects of potential treatment interventions. The computer model is expandable, so that new forms of therapies can be included.

Based on a case from an untreated patient with hepatocellular carcinoma (HCC) and multiple metastases in the liver we investigate two fundamental questions, namely whether metastases metastasize in general and secondly if late disseminated tumour cell are capable to form metastases. The results indicate that these late metastases are at least in this one case of a HCC clinically not relevant, if the patient is left untreated.

## Methods

### Mathematical Description

The computer model is based on the mathematical model by Iwata et al. [15]. Here, the parameter x describes the tumour size as the number of cells in the tumour. The primary tumour starts as a single malignant cell at the time t = 0 and growths with the rate *g(x)*. Different growth functions can be applied for *g(x)*, e. g. linear, exponential, power law or Gompertzian growth. In this work the Gompertzian growth was used since most tumours exhibit this behaviour and it fits to the clinical data in this case.

The number of cells in the tumour at time *t* is given by the function *x(t)*, which is the solution of:
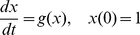
(1)Solving equation 1 with the Gompertzian growth rate for *g(x)*, the number of cells at the time *t* is given by the following function,
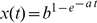
(2)where *b* is the size of the tumour at its saturated level and *a* is the growth rate constant.

The primary tumour spreads metastases with the colonization rate *β(x)*. For this rate Iwata et al. assumed the following form:
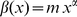
(3)where *m* is the colonization constant and *α* is the fractal dimension which describes how well the tumour is supplied with blood. The colonization rate includes only those cells that survive intravasation, the bloodstream and extravasation and are capable of founding new metastases.

It is assumed that the metastases growth with the same rate *g(x)* as the primary tumour and are also able to spread metastases with the rate *β (x)*.

### Computer Model

The computer model describes the behaviour of malignant cells in the metastatic progression as presented in detail previously [21].

**Figure 1:**
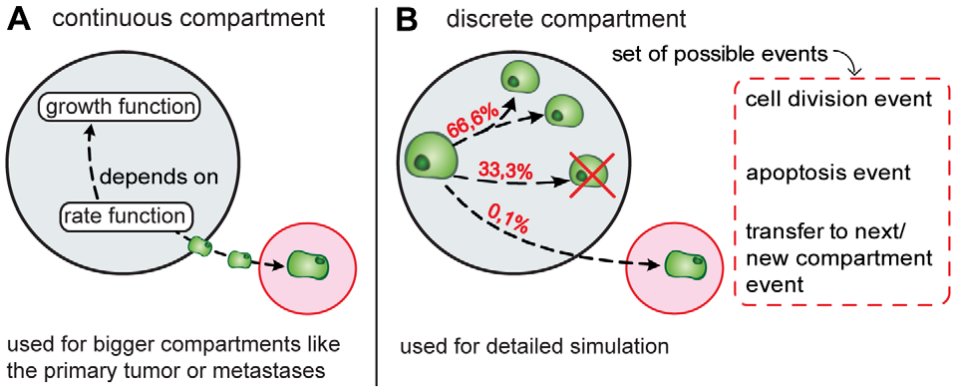
Compartment types. Compartments describe all parts that can contain malignant cells, such as primary tumour, blood stream or metastases and can be modelled in two different ways: In continuous compartments (A) all internal processes are represented by mathematical functions. The growth of the system is modelled via a growth function and the spread of metastases via a rate function. In a discrete compartment (B) all internal processes are modelled with the help of events. They describe what happens to a single cell at a specific time within the compartment. Events can be e.g. cell division, apoptosis, intravasation or the creation of a new metastasis and occur with an assigned probability in the compartment. Discrete compartments are used to simulate a compartment in detail. Continuous compartments are used to simulate bigger systems like the primary tumour or metastases.

The computer model is developed as a building kit. It provides different building blocks, from which different simulation setups can be assembled. The two most important building blocks are compartments and events. Compartments describe all organs that can contain malignant cells, such as the primary tumour, the blood vessels or the connective tissue of distant organs harbouring metastases. Compartments can be modelled either continuous or discrete (Fig. 1). In a continuous compartment all internal processes are described by mathematical functions. The growth of the primary tumour or a metastasis is represented by a growth function while the spread of metastases is represented by a colonisation rate. The parameter for the growth and colonisation function can be adapted for each continuous compartment. Even different growth and colonisation functions can be applied for different continuous compartments in this simulation setup.

In a discrete compartment all internal processes are modelled with the help of so called events. An event describes what happens to a single cell in a compartment at a specific time. Events can be cell division, apoptosis, intravasation or the creation of a new metastasis (including extravasation). A discrete compartment can be understood as a bucket, where cells can be put in and deleted from. The growth or decrease of the compartment is modelled by simulating every cell division, apoptosis, intravasation and extravasation of every cell in the compartment. Events are processed in order of the time on which they occur. After processing an event a new event is created which defines what happens next to the cell in the compartment. Every discrete compartment owns a set of possible events that can occur in this specific compartment and every kind of event in this set has a probability with which it occurs. In this way a discrete compartment can be parameterized to describe different settings, like the primary tumour, blood stream or tissues where metastases will develop.

Discrete compartments are used to simulate a compartment in detail. Since this detailed simulation is very time consuming, bigger compartments like the primary tumour or the metastases are represented by continuous compartments.

This building kit structure of the computer model allows simulation of a larger number of factors as it is possible with the analytical model of Iwata et.al. It allows changing the metastatic behaviour during time course, the simulation of the resection of the primary tumour and the assignment of different growth rates for primary tumour and metastases.

**Figure 2:**
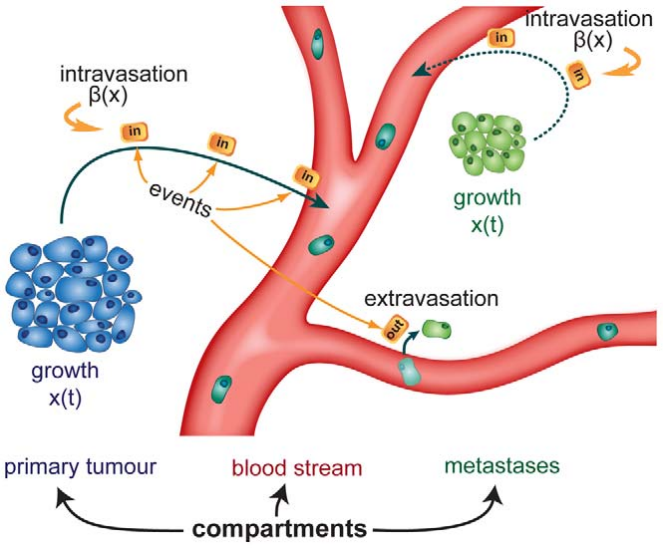
The simulation configuration used for the simulations. The growth of the primary tumour and metastases are modelled via the mathematical function *x(t)* (see eq. 2). The blood stream is modelled via events. The intravasation events are created conforming to the colonization rate *β(x)* (see eq. 3). Executing the intravasation event, a cell is added to the blood stream and a new event describing what happens next to this cell is generated. In the simulated scenarios it is examined whether metastases are able to metastasize (dotted line) and whether particularly late disseminated tumour cells are capable to form metastases.

The following configuration of compartments and events are used (see also Fig. 2):

Primary tumour and metastases are modelled as continuous compartments, the growth of both primary tumour and metastases being described by eq. 2. The spread of metastases by the primary tumour and the metastases is described by the colonization function given in eq. 3. In scenarios where metastases are not able to metastasize, a colonisation rate of zero is applied for the metastases. In the scenarios where the ability of late disseminated cells to form a metastasis is tested, the colonisation rate of the corresponding tumours is set to zero as soon as they reach a predefined size. The resection of the primary tumour is simulated by setting the growth rate and colonisation rate to zero at the day of the resection.

The blood stream is modelled as a discrete compartment. Intravasation events are created conforming to the colonization rate defined in eq. 3. Processing an intravasation event the number of cells in the bloodstream is increased by one and a new event is created, which describes the behaviour of the new cell within the bloodstream. Since the colonisation rate used only includes those malignant cells that survive in the blood stream and found new metastases, the set of possible events for the blood stream compartment includes only the extravasation event. Each surviving tumour cell in the blood stream extravasates independently into the tissue. Most cells remain in the blood for about 60 minutes but divergences of about +/−20 minutes are possible. As a consequence, the cells don't necessarily leave the blood stream in the same order they entered it. The dwelling time each cell remains in the blood stream is computed following a Gaussian distribution. The values for the mean (60 min) and the standard deviation (20 min) were determined experimentally. Similar times were published by Meng et.al. [22]. They estimated a half-life for circulating tumour cells of 1 or 2.4 hours, resp.

Each scenario was simulated 100 times. After completion of all simulations of a scenario the mean and standard deviation were computed. To compute the total tumour mass of the primary tumour and all metastases a cell mass of 10^−12^ kg [13], [17], [20] was assumed.

It is planned to make the software public available in an upcoming publication.

### Clinical Data

The simulation model was compared to clinical data from a case of a patient with hepatocellular carcinoma (HCC) [15]. Patients diagnosed with HCC have poor survival prognosis. The overall median survival after diagnosis is 10 [23] to 11 [24] month, respectively. Patients who undergo surgery or a liver transplantat have the best prognosis with a median survival between 21,6 [25] and 52 [24] months, while untreated patients have a median survival of about 2 months [23], [25]. Depending on tumour stage at the time of diagnosis, a longer survival is also possible. In particular, Okuda Stage I patients have a median survival of 8,3 months, however, also survival times up to 42 months were observed as well [25].

The patient used to model cancer progression was diagnosed while still at an early stage. The primary tumour had a size of 1,55×10^9^ cells at the time of diagnosis and no metastases were detectable. Later during the course of disease several metastases were detected in the liver. Chemotherapy was started 639 days after the first diagnosis. During the time until chemotherapy was commenced several CT scans were performed. The scans were performed at days 0 (day of the first diagnosis), 50, 89, 432, 559 and 632. The progression of the metastases can be monitored in the last three CT images. The number of metastases detected in these CT images was 10 at day 432, 28 at day 559 and 48 at day 632, respectively.

**Figure 3:**
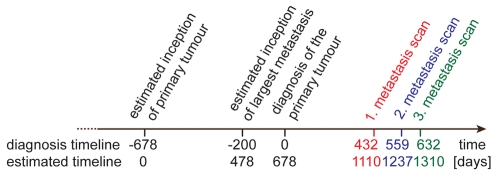
Time line of cancer progress and CT scans. The time line visualizes the progress of the cancer growth and the CT scans taken to detect metastases. The upper time dates were determined in reference to the diagnosis. In the second time line the dates were adapted in reference to the estimated origin of the primary tumour.

With the help of the mathematical model Iwata et al. estimated the day of the tumour inception to be 678 days prior the first diagnosis (Fig. 3).

The values of the four parameters of the mathematical model were determined by fitting the mathematical curves to the clinical data. The values were: *a = 0.00286 day^−1^*, b = 7.3*10^10^, m = 5.3*10^−8^ (cell day)^−1^ and *α = 0.663* [15]. The same values were used in the computer model and the simulations.

### Simulated Scenarios

**Figure 4:**
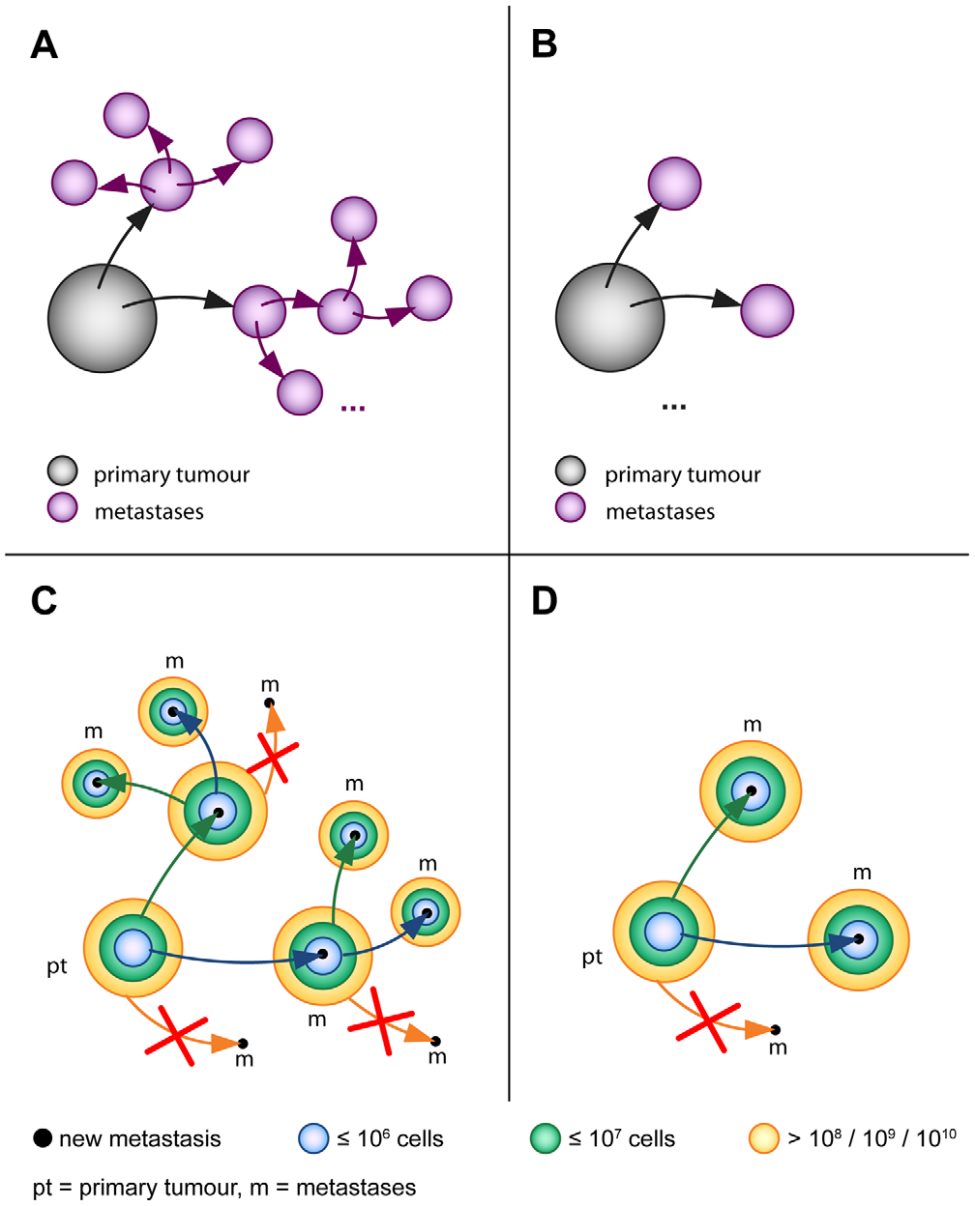
Scenarios A–D. In scenario A both primary tumour and metastases are able to spread metastases. In scenario B only the primary tumour is able to spread new metastases. In scenario C and D it is examined whether tumour cells that are disseminated late during the development of the tumour loose the capability to form metastases. Scenario C in case that metastases metastasise and scenario D in case that metastases do not metastasise. The size of the tumour is used as a benchmark to estimate the tumour development and therefore the term “late” disseminated tumour cell. Three sizes are chosen with respect to the maximum tumour size of the presented case of a HCC: 10^8^, 10^9^ and 10^10^ cells. Cells that are disseminated into the blood from the primary tumour (scn. C and D) and from metastases (scn. C) after they reached a size of 10^8^, 10^9^ or 10^10^ cells, respectively, are no longer able to form a metastasis.

Six different scenarios were examined in the simulations (Table 1). In the four scenarios A–D, which are discussed first, the ability to form metastases was investigated (Fig. 4). In scenario A both the primary tumour and metastases are able to seed metastases. In scenario B only the primary tumour is able to seed metastases. In scenario C, again, the primary tumour and metastases are both able to metastasise. However, late disseminated tumour cells lose their ability to form metastases as well. In scenario D late disseminated tumour cells lose their ability to form metastases as well, while in contrast to scenario C only the primary tumour is able to metastasise.

**Table 1:** Scenarios considered for simulation.

	metastases are able to metastasize	metastases are not able to metastasize
Late disseminated tumor cells **do not lose** the ability to form metastases.	A	B
Late disseminated tumor cells **lose** the ability to form metastases.	C	D
The primary tumor is resected.	A_R_	B_R_

Six scenarios were considered for the computer simulations. In the scenarios A and B it is tested whether metastases do metastasize or not. In the scenarios C and D it is additionally investigated whether late disseminated tumour cells might be less capable to form metastases. To define the term “late” the size of the primary tumour/metastases is used as a benchmark. In the scenarios A_R_ and B_R_ the resection of the primary tumour is simulated. Again, once for the case that metastases are able to metastasise and once for the case that metastases are not able to metastasise.

In the scenarios C and D the size of the tumour was used as a benchmark for the stage of development of the primary tumour and metastases and therefore for the definition of a late disseminated tumour cell. Three different sizes were chosen with regard to the maximum size of the presented case of a HCC (7.3*10^10^ cells). The chosen values are 10^8^, 10^9^ and 10^10^ cells (Fig. 4, panels C and D). All cells that were disseminated from tumours after they reached the corresponding size were no longer able to generate new metastases and eventually died in the bloodstream. Hence, these cells had no impact on the further course of the simulated system. To save simulation time, only those cells that survived in the bloodstream and formed a metastasis were considered. Therefore, the process of intravasation and apoptosis of late disseminated cells was condensed by setting the colonisation rate to zero after the tumours reached the critical size of 10^8^, 10^9^ and 10^10^ cells, resp. It is highly unlikely that in reality the colonisation rate will abruptly drop from its actual value down to zero as it is set in these scenarios. To our knowledge there are no experimental or clinical data available that describe the dropping of the dissemination rate. Therefore, both extreme cases (no dropping: scn. A and B, instant dropping: scn. C and D) were chosen to compare the effects of both extreme cases.

In the scenarios A_R_ and B_R_ a possible treatment, the resection of the primary tumour, was simulated. In scenario A_R_ primary tumour and metastases were able to metastasize, whereas in scenario B_R_ only the primary tumour spreads metastases. For both scenarios two different time points were chosen for the resection. The first time point was two days after the first diagnosis, at day 680. To analyse how the fate of the patient would have developed if the carcinoma had been diagnosed earlier, the second time point was set about two months before the first diagnosis, at day 620. At this day the primary tumour had a size of 1,044*10^9^ cells (∼1 g), which is about the minimum size for clinical detection of a primary tumour.

As an approximate benchmark for comparing the different scenarios in relation to the relevance for the patient, the total tumour mass of the primary tumour and all metastases was used. For HCC different staging systems exist, to predict the prognosis of patients [26]. These staging systems include different factors like the tumour size, the presence of metastases, the liver function or the general health status of the patient. Since the computer simulations do not include information about the liver function and the general health of the patient and additionally the staging systems are very vague about the tumour size and number of metastases in end stage HCC, the total tumour mass was used as a reference value to predict the survival time of the patient. The value of 1 kg serves as a benchmark for the total tumour mass [27]. At this approximate size the cancer leads typically to organ failure or several systemic processes that cause the patient's death [13]. This value of 1 kg refers to tumours that also spread metastases in organs other than the primary tumour. In the presented case of a HCC the metastases remain only in the liver, which implies that the lethal tumour mass might be smaller than 1 kg. But since the exact value is not known for a HCC, the value of 1 kg was set to compare the different scenarios, while eventually smaller or larger tumour burden could cause the death of a patient as well.

## Results

### Existing clinical data of this case are not sufficient to determine if metastases are able to metastasize or not

As described in the introduction section it remains unclear, if metastases are able to metastasize or not. In order to answer this question, scenarios A and B were simulated in the computer model. The graphs A and B in Fig. 4 show the cumulative number of the metastases dependent on the size for the two scenarios, respectively. The corresponding standard deviations for both scenarios are shown in Fig. S1 and S2. The size of the metastases is represented by the number of cells. The graph shows the number of metastases of the size equal or greater to the chosen value on the x-axes. The cumulative histograms were computed for the three days on which the CT scans detected the metastases. The simulation results of both scenarios show no difference in the range of the clinical data. Only late in time course and for very small metastases, i. e. smaller than 10^6^ cells the graphs differ noticeably (Fig. S3). Hence, based on this clinical data it is not decidable whether in this case of a HCC the metastases were able to metastasize or not.

### Late disseminated tumour cells are capable to form metastases

As mentioned in the introduction section it is unknown whether tumour cells disseminated late during the development of the primary tumour and metastases are still capable to form further metastases. This question was examined in the modelled scenarios C and D (Table 1, Fig. 4 panels C and D). In both scenarios cells that were disseminated from the primary tumour (scn. C and D) and metastases (scn. C) after the tumours reached a size of 10^8^, 10^9^ or 10^10^ cells, resp., were no longer able to form metastases.

**Figure 5:**
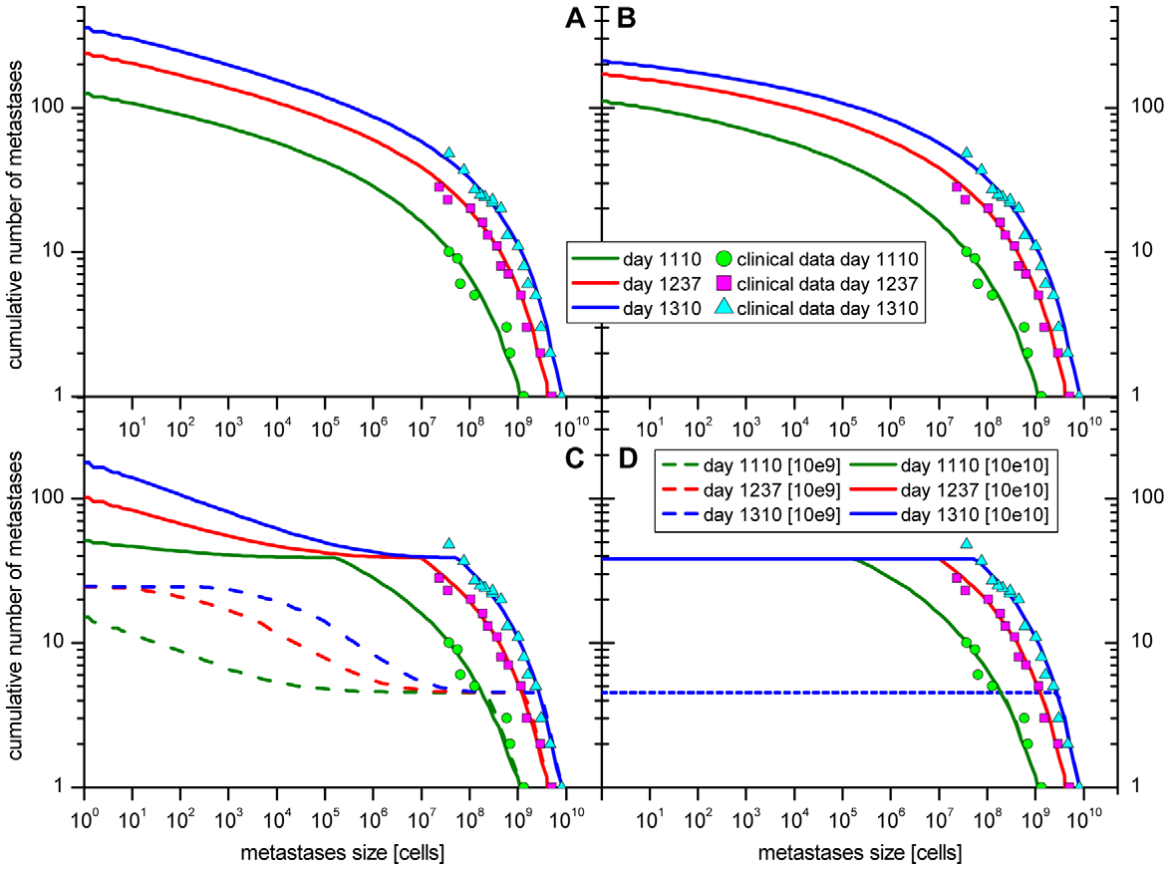
Case of a hepatocellular carcinoma with multiple metastases in the liver. The circles, squares and triangles represent the clinical data taken from the patient at the days 1110, 1237 and 1310 after the estimated origin of the primary tumour. The cumulative number of metastases according to the size of the metastases is shown. Four different scenarios were simulated (see Table 1 and Fig. 4). The results for the scenarios A and B both fit well with the clinical data. The simulated data differs only in the range of very small metastases (see also Fig. S3). Hence, on the basis of the available clinical data it is not decidable whether metastases metastasise or not. The simulation results of scenarios C and D clearly show a plateau, which arises from the fact that the primary tumour reached the plateau size of 10^9^ (dashed lines) or 10^10^ (solid lines) cells, respectably. All cells that are disseminated from the primary tumour from now on are no longer able to form a metastasis. In scenario D only the primary tumour is able to metastasise, so no further metastases are created. The number of metastases stays the same while the existing metastases keep on growing. In scenario C metastases are able to spread new metastases. As a consequence the number of metastases start rising again, as soon as the first metastases spread from the primary tumour are large enough to spread metastases themselves. A second plateau is discernible for the case that cells disseminated from tumours larger than 10^9^ cells lose the ability to form metastases (dashed lines). This second plateau indicates that the first metastasis reached the size of 10^9^ cells, too. Such a second plateau cannot be observed for the solid lines since up until day 1310 none of the metastases reached the critical size of 10^10^ cells. In addition to this figure the frequency distribution of the metastases sizes was plotted in Fig. S7.

The results of the simulations are shown in Fig. 5, panels C and D. The corresponding standard deviations for both scenarios are shown in Fig. S4 and S5. Under the assumption that tumour cells are no longer able to form metastases if they were disseminated from a primary tumour larger than 10^8^ cells, no metastases occur at all. Therefore, no corresponding data are shown in the graphs. Next, it was investigated whether disseminated tumour cells are no longer capable to form metastases if they disseminated from the primary tumour or a metastasis larger than 10^9^ cells. Comparing the simulation results for this scenario (dashed lines in Fig. 5, panels C and D) with the clinical data, it is obvious that the simulated data does not fit to the clinical reality. Only the data points of the three biggest metastases of each CT scan fit to the simulation results, indicating that cells that were disseminated from the primary tumour or a metastasis larger than 10^9^ cells indeed keep their ability to form a metastasis. Finally, it was examined whether tumour cells that were disseminated from the primary tumour or metastases larger than 10^10^ cells were unable to form metastases. The simulation results of this scenario (solid lines in Fig. 5, panels C and D) were compatible with the clinical data, indicating that it might be possible that cells that were disseminated from the primary tumour or metastasis larger than 10^10^ cells were not longer able to form metastases. But since the clinical data did not cover the whole range of metastases sizes, it might also be possible that they keep the ability to form a metastasis after the tumour had reached this size. As in the comparison of scenario A and B, the simulation results of the scenario C (metastases do metastasise) and D (metastases do not metastasise) show no differences within the range of the clinical data. Differences were only seen late during time course and for very small metastases (<10^6^ cells).

### Significant differences in the number of metastases

**Figure 6:**
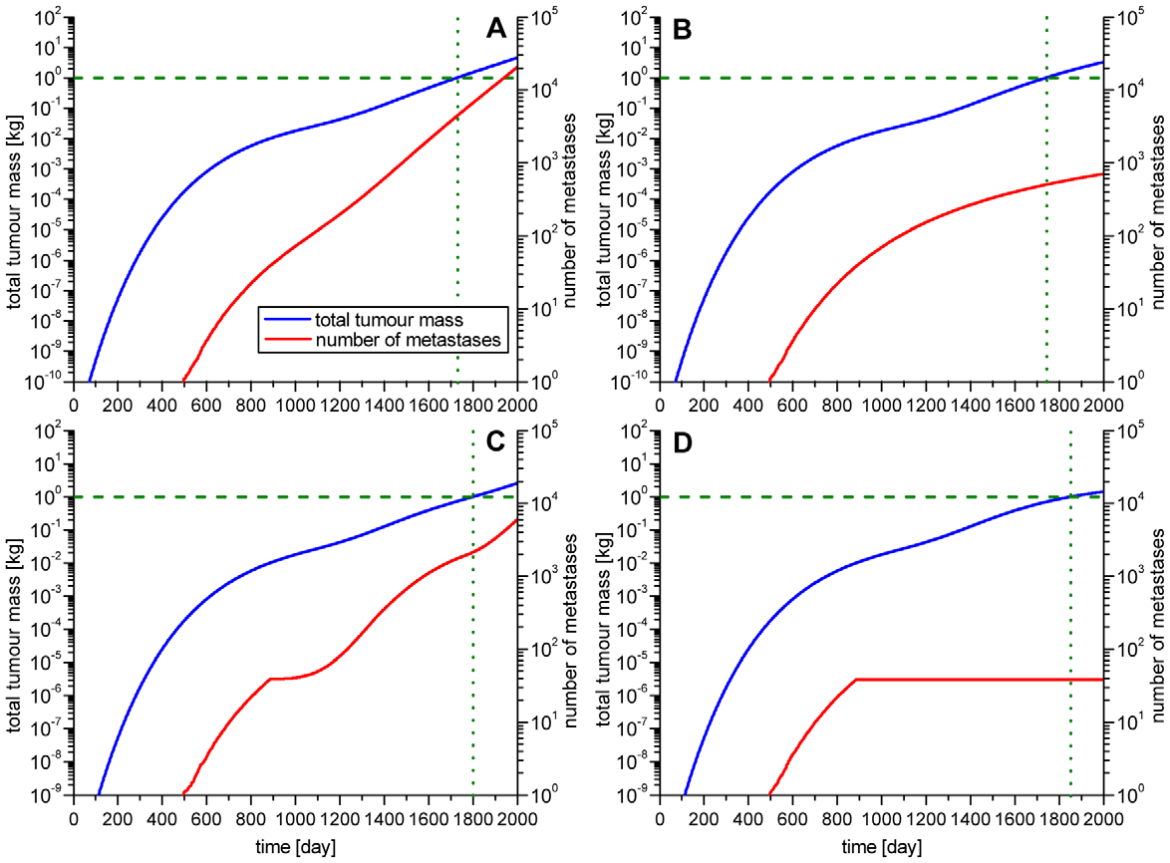
The development of total tumour weight and number of metastases in time. To compare the different scenarios in terms of relevance for the patient the total tumour mass (blue line), including the primary tumour and all metastases, was computed. The value of the lethal tumour mass of 1 kg is marked with a dashed line. The number of metastases (red line) was also visualized in the graph. The tumour mass is mapped to the left y-axes and the number of metastases to the right y-axes. The day on which primary tumour and metastases reach the total mass of 1 kg is marked with a green dotted line. Again four different scenarios were simulated: A) Metastases are able to metastasize. B) Metastases are not able to metastasize. C) Primary tumour and metastases metastasize only until they reach a size of 10^10^ cells. Metastases are able to metastasize. D) Primary tumour and metastases metastasize only until they reach a size of 10^10^ cells. Metastases are not able to metastasize.

Figure 6 shows the development of the total tumour mass and the number of metastases during the course of the disease. As described above the value of 1 kg serves as a benchmark for comparing the different scenarios in relation to the relevance for the patient. The day on which the total tumour mass of 1 kg is reached is marked with a dotted line. The number of metastases at this time point significantly varied between the four scenarios A–D (see Table 2). Especially between the scenarios where metastases are able to metastasize (A and C) and where they are not (B and D) the differences were very large. In scenario A 4402 metastases and in scenario B 501 metastases existed at the day the total tumour mass of 1 kg was reached, leading to a total difference of 3901 metastases. In scenario C the number of metastases is 2131, while in scenario D the number of metastases at the day the total tumour mass of 1 kg is reached is 38, leading to a difference of 2093 metastases.

**Table 2:** Number of metastases on the day of achieving a total tumour mass of 1 kg.

	No Treatment				Resection			
Scenario	A	B	C	D	A_R_		B_R_	
Day of resection	---	---	---	---	680	620	680	620
Day of reaching lethal tumour mass	1728	1740	1800	1852	1972	2018	---	---
Number of metastases on that day	4402	501	2131	38	4658	4276	---	---

The day of reaching a total tumour mass of 1 kg varies from day 1728 in scenario A to day 1852 in scenario D. By resecting the primary tumour this day can be shifted further into the future. In scenario A_R_ the lethal tumour mass is reached at day 1972 if the primary tumour is resected at day 680 or at day 2018 if the primary tumour is resected at day 620. In scenario B_R_ the tumour mass of 1 kg is no longer reached. The significant differences in the number of metastases between the scenarios where metastases are able to metastasize and the scenarios where they are not are particularly remarkable. The difference between scenario A and B is 3901 metastases and between the scenarios C and D 2093 metastases. In contrast, the day of reaching a total tumour mass of 1 kg was only shifted by 12 or 52 days, respectively, into the future.

### Total tumour mass is unaffected if metastases do metastasize or not

In contrast to the large differences in the number of metastases, the time reaching a total tumour mass of 1 kg differed only very little. In scenario A it is reached at day 1728 while in scenario B it is reached at day 1740. This accounts for a difference of only 12 days. In contrast the number of metastases varied about 3901 metastases. In the scenarios C and D the day of reaching a total tumour mass of 1 kg was shifted into the future only by 52 days, while the number of metastases varied about 2093 metastases.

### Metastasis formation is an early event

As already briefly shown in the mathematical analysis of the patient's data, the computer simulations showed that in this case of a HCC metastasis is an early event. In the computer simulations the first metastasis was seeded at day 496. At this time point the primary tumour had a size of 1.71286*10^8^ cells, which equals to 0.17129 g. At this size the primary tumour is clinically not detectable, which makes metastasis formation a very early event.

### Resection of the primary tumour delays the time of death

**Figure 7:**
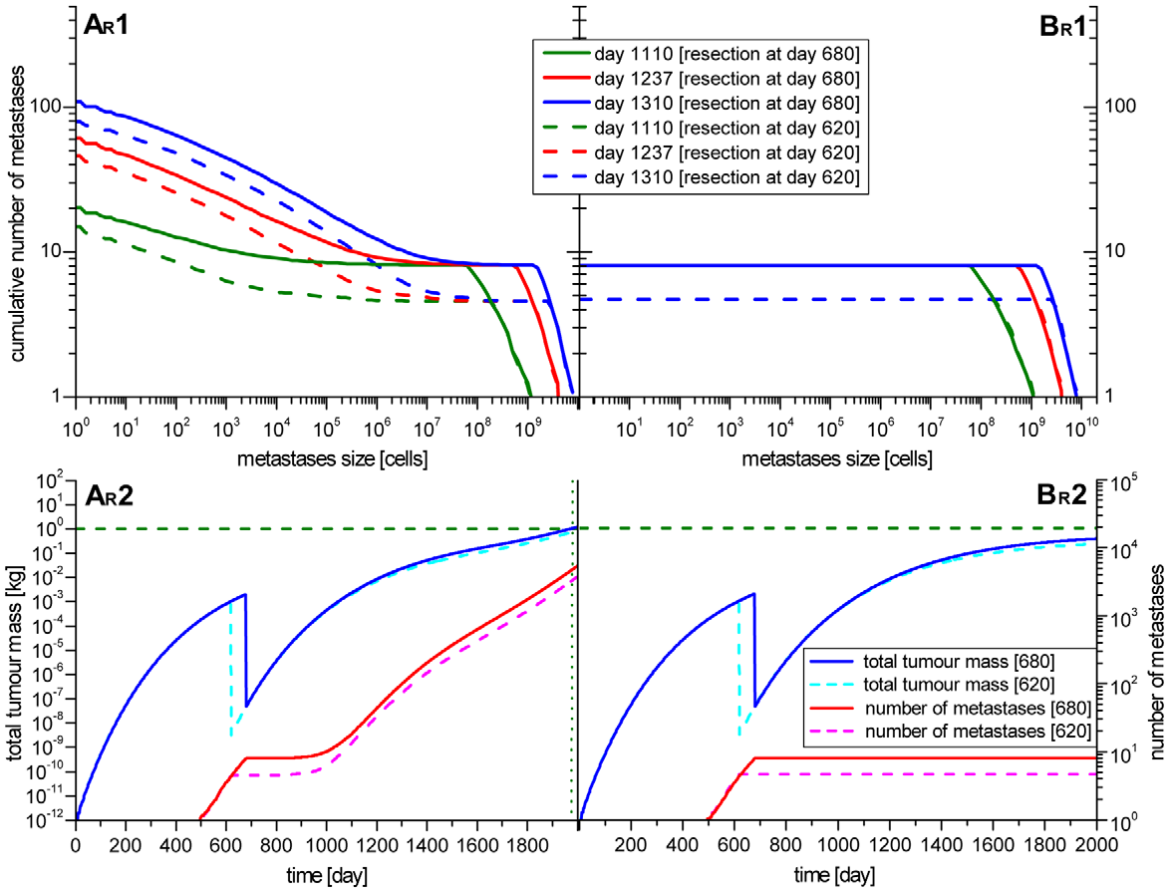
Simulated resection of the primary tumour. The resection of the primary tumour was simulated for two different time points. The first time point is shortly after the initial diagnosis at day 680 (solid lines). The second time point is some time before the first diagnosis at day 620 (dotted lines) simulating early diagnosis e.g. as a result of tumour screening. The cumulative number of metastases according to the size of the metastases (graphs A_R_1 and B_R_1) and the development of the tumour mass and the number of metastases in time (graphs A_R_2 and B_R_2) are shown. After the resection no new metastases are founded for some time. In scenarios A_R_ the number of metastases starts rising again after some time. The time of death could be shifted to 244 days (resection at day 680) or 290 days (resection at day 620) into the future. In scenario B_R_ no new metastases are founded after the resection. The existing metastases keep growing, but never reach the benchmark value of 1 kg.

To test the relevance of resection of the primary tumour on metastasis formation, the scenarios A_R_ and B_R_ were examined. Here the resection of the primary tumour was simulated for two different points in time. Once, two days after the diagnosis and secondly around two months before the first diagnosis, when the primary tumour had reached the smallest detectable size of about 1 g (10^9^ cells). The simulation results of both scenarios are shown in Fig. 7.

In scenario A_R_ the metastases were able to metastasize. After the resection of the primary tumour the foundation of new metastases stagnated, seen as plateaus in the graphs A_R_1 and A_R_2 of Fig. 7. After a time span of about 280 days (resection at day 680) or 350 days (resection at day 620), respectively, the number of metastases eventually increased again. This increase can be explained by the fact that the metastases that were seeded before the resection of the primary tumour are first too small to spawn new metastases. After they have grown to a certain size they start emitting malignant cells and therefore are able to seed new metastases. Although the creation of new metastases could not be stopped, the day of reaching the lethal tumour mass shifted from 244 days (resection at day 680) to 290 days (resection at day 620), respectively, into the future. Thus, the survival time after diagnosis is 43.1 or 44.7 month, respectively, which fits well with clinical observations [23]–[25] and is a clearly noticeable improvement for the patient.

The life elongating effect is even stronger in scenario B_R_. In this scenario metastases were not able to metastasize. Therefore no new metastases were seeded after the resection of the primary tumour, since only the primary tumour was able to seed metastases. The existing metastases keep on growing but are unable to seed new metastases. After the resection only 8 (resection at day 680) or 4 (resection at day 620) metastases remain in the body of the patient. According to the mathematical model from Iwata et al. (see above), metastases grow with a Gompertzian rate. This implies that they cannot grow indefinitely but stagnate at a specific time point. In this particular case of HCC Iwata assumed a stagnation size of about 7.3*10^10^ cells. Hence, since only 8 or 4 metastases are left, this stagnation size would lead to the result that the lethal tumour mass of 1 kg was never reached in this scenario. However, this result is unlikely to reflect the clinical situation of the patient (see discussion).

## Discussion

### In hepatocellular carcinoma metastases exhibit the same growth rate as the primary tumour

The mathematical model assumes that primary tumour and metastases grow with the same rate and seed metastases with the same rate. Therefore, in the computer simulations the metastases were parameterized with the same growing and colonization behaviour as the primary tumour. Comparing the simulation results and the clinical data (panels A and B in Fig. 4), it seems to be correct for this particular case of a HCC. This result seems to be plausible since the primary tumour is growing fast and the metastases are located in the same organ as the primary tumour. Further simulations with a higher growth rate for the metastases (+50% and +100% of the growth rate of the primary tumour) support this assumption (Fig. S6).

This HCC is a special case of metastasis formation, as metastases grow in the same organ as the primary tumour does. It is most likely that this metastasis pattern does not hold true for all cases of HCC and all kinds of cancers, especially if metastases grow in other organs than the primary tumour. In other organs different growth conditions might apply. It is very likely that in this cases growth rates will differ between the primary tumour and metastases and probably even between metastases in different organs, since the microenvironment of the various organs supports or hampers the growth of metastases differently for different kinds of cancer as described below.

### Only a small amount of metastases is responsible of the patients death

The results revealed significant differences in the number of metastases between scenarios where metastases were able to metastasize and scenarios where they were not. Surprisingly, the time span until a total tumour mass of 1 kg is reached differs very little between these two scenarios indicating that this question is biologically and clinically insignificant for the patients' outcome.

This little gain in survival is in contrast to the large differences in the number of metastases, indicating that small metastases are of no clinical relevance for the patient. In scenario D 38 metastases are enough to reach a total tumour mass of 1 kg. Hence, not the total amount of metastases is responsible for the patient's death but the first metastases that were able to grow into clinically detectable macro-metastases.

### The simulation of other cancer types promises different results

The simulation results were validated with clinical data from a fast growing HCC with metastases in the liver only. The computer model was parameterized to describe this particular case of a HCC. The results cannot easily be transferred to other carcinomas, since several studies showed that the distribution of metastasis to different organs varies between different kinds of carcinomas, the seed and soil hypothesis [28]–[30] or even between different subtypes of one carcinoma entity [31]. Most carcinomas have a typical set of organs to which they metastasize. These patterns are due to vascular pathways and the microenvironment in the organs the disseminated cells end up. Molecular factors like growth factors, cytokines and signalling cascades influence the outgrowth of metastases in a particular organ [32]. Further mutations at the new site may also lead to adaption and therefore to genetic disparity between the metastases and the primary tumour and even between metastases in disparate host organs [33].

So, in a next step the computer model should be applied to other malignancies, e.g. breast cancer, or other relatively slow growing tumours, or fast growing malignancies like melanoma, to test whether the drawn conclusions can be generalized or if not, which different conclusions can be drawn.

In principle, the computer model is already able to model these different situations as it allows applying different growth rates for primary tumour and metastases and even different kinds of metastases. It also allows defining different probabilities of spreading into different organs like lung, brain or bone marrow. However, these modalities will be described in a further communication.

### Stagnation size of the primary tumour is error-prone

The simulation results of scenario B_R_ can lead to the assumption, that the patient would not die, if metastases are unable to metastasise and the primary tumour had been resected. This assumption has to be considered carefully. First, the value of 1 kg represents a rough benchmark for comparing different scenarios. In the presented case of a HCC the metastases remained in the liver only, which implies that the lethal tumour mass might actually be smaller than 1 kg. The actual value might depend on the liver function and the general health of the patient.

Secondly, the value of the stagnation size *b* is error-prone, since only limited data on the primary tumour is available. The available data indicates that the primary tumour is still in the fast growing stage of the Gompertz growth and that the detected metastases were far from the estimated stagnation size of 7.3*10^10^ cells. The biggest metastasis measured in all three CT scans had a size of 8.12*10^9^ cells. With this limited data the determination of the stagnation size *b* via fitting the theoretical curve to the observed data is error-prone. Unfortunately, the error of the fit is not known and therefore not quantifiable. However, computer simulations with a varied stagnation size *b* show that higher values of e.g. +10% (8.08*10^10^ cells) or +20% (8.76*10^10^ cells) are still compatible with the observed clinical data (data not shown here).

So, assuming a lower lethal tumour mass and a higher stagnation size of the primary tumour and metastases it may still be possible that the patient dies after the resection of the primary tumour for the scenario that metastases were not able to metastasise.

### More detailed clinical data including smaller metastases required

As described in the results section the available clinical data is not detailed enough to decide whether metastases metastasise or whether they do not. Within the range of the clinical data the graphs do not differ (Fig. 5, panels A and B and Fig. S3).

Tumours start to metastasize from a certain minimal size onwards. Therefore the effect whether metastases do metastasize can only be observed, after the first metastasis had enough time to grow and spread itself. Hence, no single value can be attributed for the size of metastases at which the two scenarios A and B can be clearly distinguished.

Comparing not only the mean but also the standard deviation, it is nearly impossible to clearly distinguish between both scenarios at day 1110. At day 1237 the two scenarios can be separated in the metastasis size range of 1 till 1000 cells. At day 1310 this range broadens up till about 10^4^ cells. Neglecting the standard deviation and only comparing the mean values of both scenarios, the range shifts as follows: day 1110→1–100 cells, day 1237→1–10^5^ cells, day 1310→1–10^6^ cells (see Fig. S3).

The question of the capability of late disseminated tumour cells to form metastases is also not completely answered. Based on the available clinical data it can be concluded that late disseminated tumour cells are at least capable to form metastases until the primary tumour (scn. C and D) or a metastasis (scn. C) reached the size of 10^10^ cells (Fig. 5, panels C and D). Whether cancer cells disseminated beyond this stage of development loose the capability to form metastases or not, cannot be decided since the clinical data is not detailed enough.

To answer these questions, additional data of the presence of metastases at least smaller than 10^6^ cells would be necessary. Therefore future studies addressing these questions should include the analysis of smaller metastases.

### The computer model can help to understand the metastatic progression

The aim of the computer model is to facilitate a better understanding of the processes of metastasis formation. In particular, this computer model allows to analyse different scenarios derived from clinical and experimental observations. Comparing the modelled data with clinical data, it might help to decide which pattern of metastatic spread applies to the clinical data.

To be able to do this, it has to be ensured that the computer model works in accordance with the clinical situation. Therefore the simulation data was validated with clinical data from a patient with HCC. The graphs A and B in Fig. 4 show that the simulated data fits quite well with the clinical data and thereby prove that the computer model works in accordance with the biology of the HCC. Based on this finding, different scenarios can be simulated, like the variation of the metastatic behaviour or different treatment modalities. Since all these scenarios can be simulated with the same model, it allows a better comparability of these scenarios. Such a comparison, as described in this paper, revealed that in this case of a HCC metastases from metastases are not clinical relevant if the tumour is left untreated and that also late disseminated tumour cells are still capable to form metastases.

The capability to simulate different kinds of scenarios is the particular advantage of the computer model used in this paper. It was not only designed for a specific part of the metastatic progression or for a single scenario of the metastatic cascade. On the contrary, the model can be easily adapted to other aspects of the metastatic cascade by changing individual parameters or by changing the growth and colonization functions. This approach would allow modelling the entire metastatic cascade for many different tumour entities. In contrast to analytical models such as described previously [15] these functions can be easily replaced without recalculating the entire model. It can furthermore be applied even if no analytical solution exists.

The development of the computer model is still at the beginning. The model and the software environment is constantly enhanced and improved but it is already capable to describe a variety of different types of malignancies.

The next step is to apply the computer model to other cancer entities and to compare the simulation results with detailed clinical data, in order to unravel the underlying important biological steps in cancer metastasis.

## Supporting Information

Figure S1 **Simulation results of scenario A with mean and standard deviation.** In scenario A primary tumour and metastases are both able to metastasise. The graph shows the cumulative number of metastases in relation to the metastasis size. The thick lines represent the mean for the three days 1110 (green), 1237 (red) and 1310 (blue). The thin black lines above and beneath each thick line display the standard deviation. The circles, squares and triangles represent the clinical data taken from the patient at the days 1110, 1237 and 1310. As can be seen, the clinical data fits well with the simulation results.
(TIF)

Figure S2 **Simulation results of scenario B with mean and standard deviation.** In scenario B only the primary tumour is able to spread metastases. As in Fig. S1, the graph shows the cumulative number of metastases in relation to the size of metastases. The thick lines represent the mean for the three days 1110 (green), 1237 (red) and 1310 (blue). The black lines above and beneath each thick line display the standard deviation. The circles, squares and triangles represent the clinical data taken from the patient at the days 1110, 1237 and 1310. Similar to scenario A (Fig. S1) the clinical data fits well with the simulation results.
(TIF)

Figure S3 **Comparison of scenario A and B.** In this figure the simulation results of the scenarios A and B are displayed in the same graph. The thick lines represent the mean and the dashed lines the corresponding standard deviations. The graph clearly shows that in the range of the clinical data, the two scenarios A and B are nearly identical. Only in the range of the smaller metastases and late during the time course the scenarios can be separated. This is plausible, since tumours have to reach a certain minimal size, before they start metastasizing. Therefore, the effect whether metastases do metastasize can only be observed, after the first metastasis had grown large enough to start spreading metastases of its own. Including the standard deviation it is nearly impossible to clearly separate both scenarios at day 1110. At day 1237 both scenarios can be distinguished only for very small metastases from the size of 1 till 1000 cells. At day 1310 both scenarios can be clearly distinguished in the metastasis size range from 1 till 10^4^ cells. If only the mean values are compared, the metastasis size ranges where both scenarios can be separated change as follows: day 1110→1–100 cells, day 1237→1–10^5^ cells, day 1310→1–10^6^ cells (see Fig. S3).
(TIF)

Figure S4 **Simulation results of scenario C with mean and standard deviation.** In scenario C both primary tumour and metastases are able to spread metastases. Cells that are disseminated from the primary tumour and from metastases after they reached a size of 10^9^ cells (dashed lines) or 10^10^ cells (solid lines), respectively, lose their ability to form further metastases. The thick lines represent the mean values, while the thin lines above and beneath each thick line represent the corresponding standard deviation. The clinical data does not fit with the dashed lines, which indicates that cells that are disseminated from tumours larger than 10^9^ cells are still able to form new metastases. In contrast, the clinical data fits well with the solid lines. This finding supports the assumption that cells that are disseminated from tumours larger than 10^9^ cells may lose the ability to form metastases. However, the clinical data is not detailed enough to definitively decide this question. Clinical data from metastases smaller than 10^7^ cells would be necessary to answer this question. The plateaus are caused by the primary tumour that reached the critical size of 10^9^ or 10^10^ cells, resp. As long as the first metastases spread by the primary do not reach the minimal size to spread metastases of their own, no new metastases are created, which explains the plateau observed. After these metastases start spreading metastases themselves, the plateau dissolves and the number of metastases starts rising again. As soon as the first metastases reach the critical size, too, a second plateau starts to form which can be observed for the dashed lines (critical size = 10^9^ cells).
(TIF)

Figure S5 **Simulation results of scenario D with mean and standard deviation.** In scenario D the metastases are not able to metastasise. Similar to scenario C it is investigated whether cells that are disseminated from the primary tumour and metastases after they reach a size of 10^9^ cells (dashed lines) or 10^10^ cells (solid lines), respectively, lose their ability to form metastases. The thick lines represent mean values, while thin black lines above and beneath each thick line represent the corresponding standard deviation. As in scenario C the clinical data does not fit with the dashed lines but with the solid lines. In contrast to scenario C the observed plateau remains, since only the primary tumour is able to metastasise. So, as soon as the primary tumour reaches the critical size of 10^9^ or 10^10^ cell, resp, no new metastases are created.
(TIF)

Figure S6 **Simulation results of scenario A and B with higher growth rate for the metastases.** The graphs show the simulation results for the scenarios A (metastases are able to metastasise) and B (metastases do not metastasise) with a varied growth rate for the metastases. The solid lines represent the simulation results for the case that primary tumour and metastases grow with the same growth rate of *a = 0.00286 day^−1^*. The dashed and dotted lines display the simulations results for the case that metastases grow with a 50% (a = *a = 0.00429 day^−1^*) or 100% (a = *a = 0.00572 day^−1^*), resp., higher growth rate than the primary tumour. The comparison of the simulation results with the clinical data clearly shows that in this case of a HCC metastases do not grow faster than the primary tumour, but that they in fact grow with the same growth rate as the primary tumour.
(TIF)

Figure S7 **Frequency distribution of scenarios A–D.** The graphs display the number of metastases belonging to the size ranges applied to the x axis. In scenario A much more new created metastases were present than in scenario B, indicating how much impact metastasising metastases gain on the number of metastases. In scenario B much fewer new metastases were created than in scenario A, since only the primary tumour was able to metastasise. The higher number of metastases for the bigger metastases sizes at the days 1237 and 1310 in scenario B results from the logarithmical division of the metastases size ranges. The graphs C_1_ and C_2_ clearly display the decrease of new created metastases after the primary tumour reached the critical size of 10^9^ (C_1_) or 10^10^ (C_2_) cells, respectively, and how the number of new metastases slowly starts rising again after the first metastases started spreading metastases of their own. In graph C_1_ a second decrease of new created metastases can be observed for the days 1237 and 1310. In contrast to the first decrease, the second decrease occurs less sudden. This happens because at the time the first metastasis reached the critical size of 10^9^ cells already multiple metastases are able to spread metastases. In contrast to scenario C only the primary tumour was able to spread metastases in scenario D. As a result no new metastases were created after the primary tumour reached the critical size of 10^9^ (D_1_) or 10^10^ (D_2_) cells, respectively. This fact can be observed in the graphs D_1_ and D_2_. The existing metastases kept on growing, but no new metastases are created. The total number of metastases is the same for the three time points (4 in D_1_ and 38 in D_2_).
(TIF)
