# Supplementary figures and images for: Are Metastases from Metastases Clinical Relevant? Computer Modelling of Cancer Spread in a Case of Hepatocellular Carcinoma
Source: PLoS One. 2012 Apr 23;7(4):e35689. doi: 10.1371/journal.pone.0035689 (PMC3335074; doi:10.1371/journal.pone.0035689)

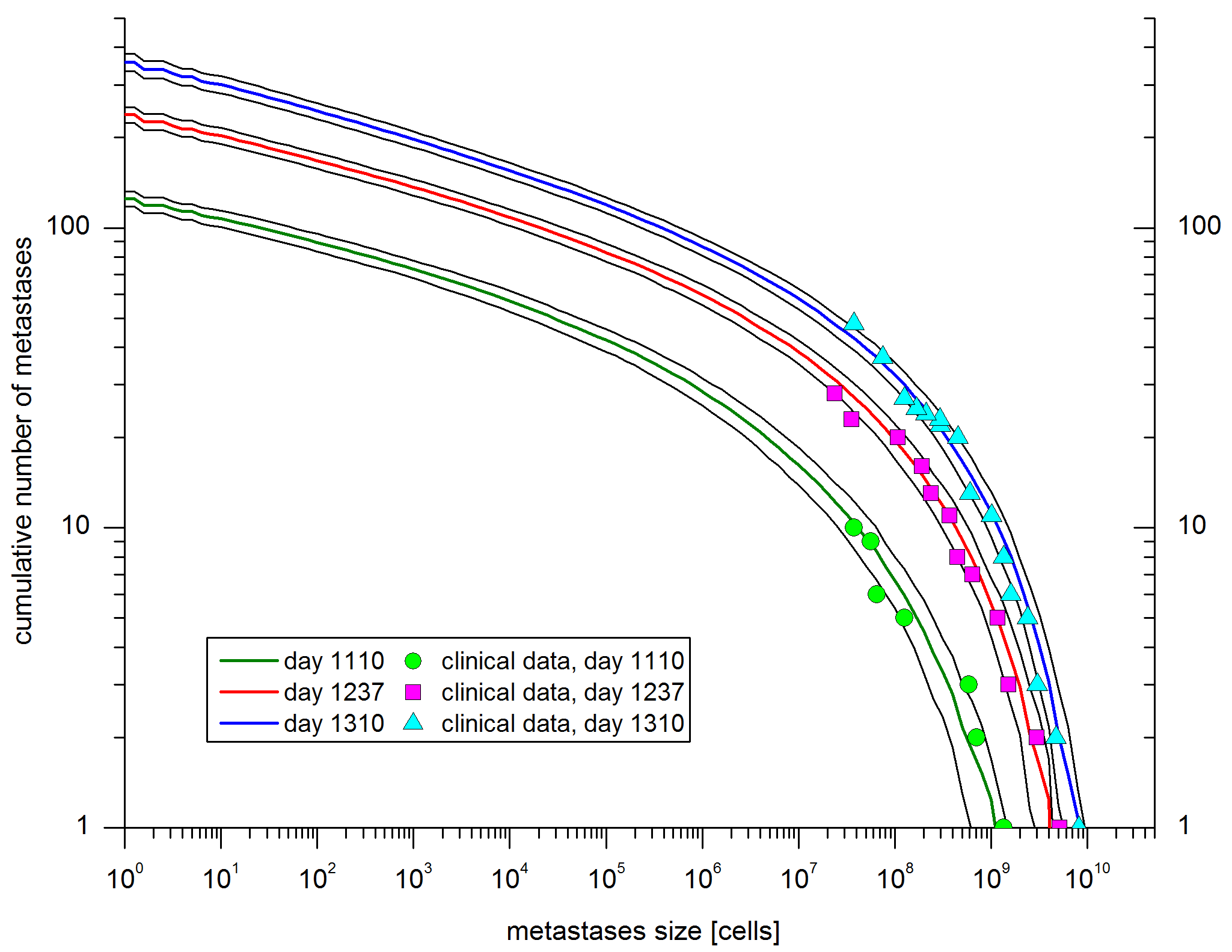

Supplement: Figure S1 — Simulation results of scenario A with mean and standard deviation. In scenario A primary tumour and metastases are both able to metastasise. The graph shows the cumulative number of metastases in relation to the metastasis size. The thick lines represent the mean for the three days 1110 (green), 1237 (red) and 1310 (blue). The thin black lines above and beneath each thick line display the standard deviation. The circles, squares and triangles represent the clinical data taken from the patient at the days 1110, 1237 and 1310. As can be seen, the clinical data fits well with the simulation results. (TIF) [file pone.0035689.s001.tif]

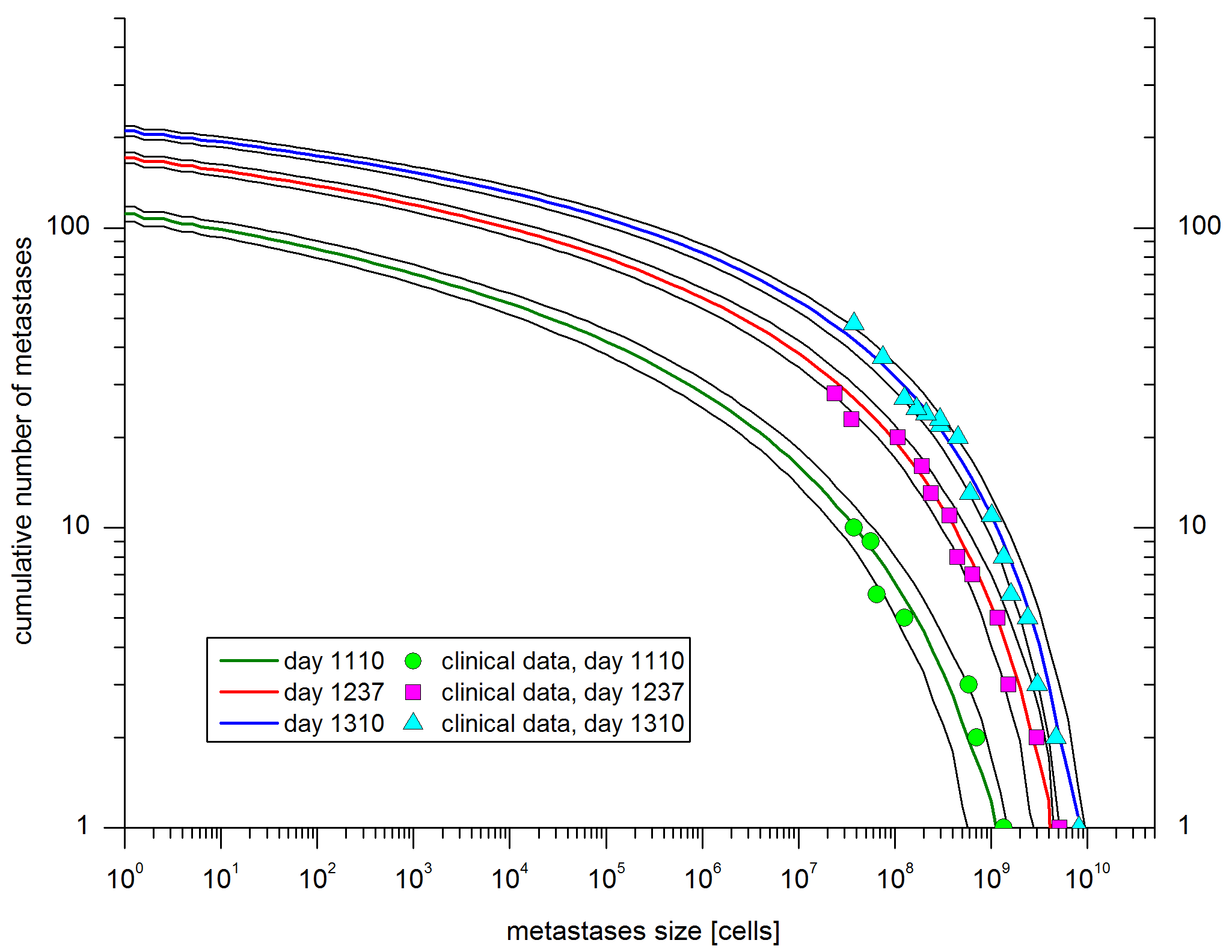

Supplement: Figure S2 — Simulation results of scenario B with mean and standard deviation. In scenario B only the primary tumour is able to spread metastases. As in Fig. S1, the graph shows the cumulative number of metastases in relation to the size of metastases. The thick lines represent the mean for the three days 1110 (green), 1237 (red) and 1310 (blue). The black lines above and beneath each thick line display the standard deviation. The circles, squares and triangles represent the clinical data taken from the patient at the days 1110, 1237 and 1310. Similar to scenario A (Fig. S1) the clinical data fits well with the simulation results. (TIF) [file pone.0035689.s002.tif]

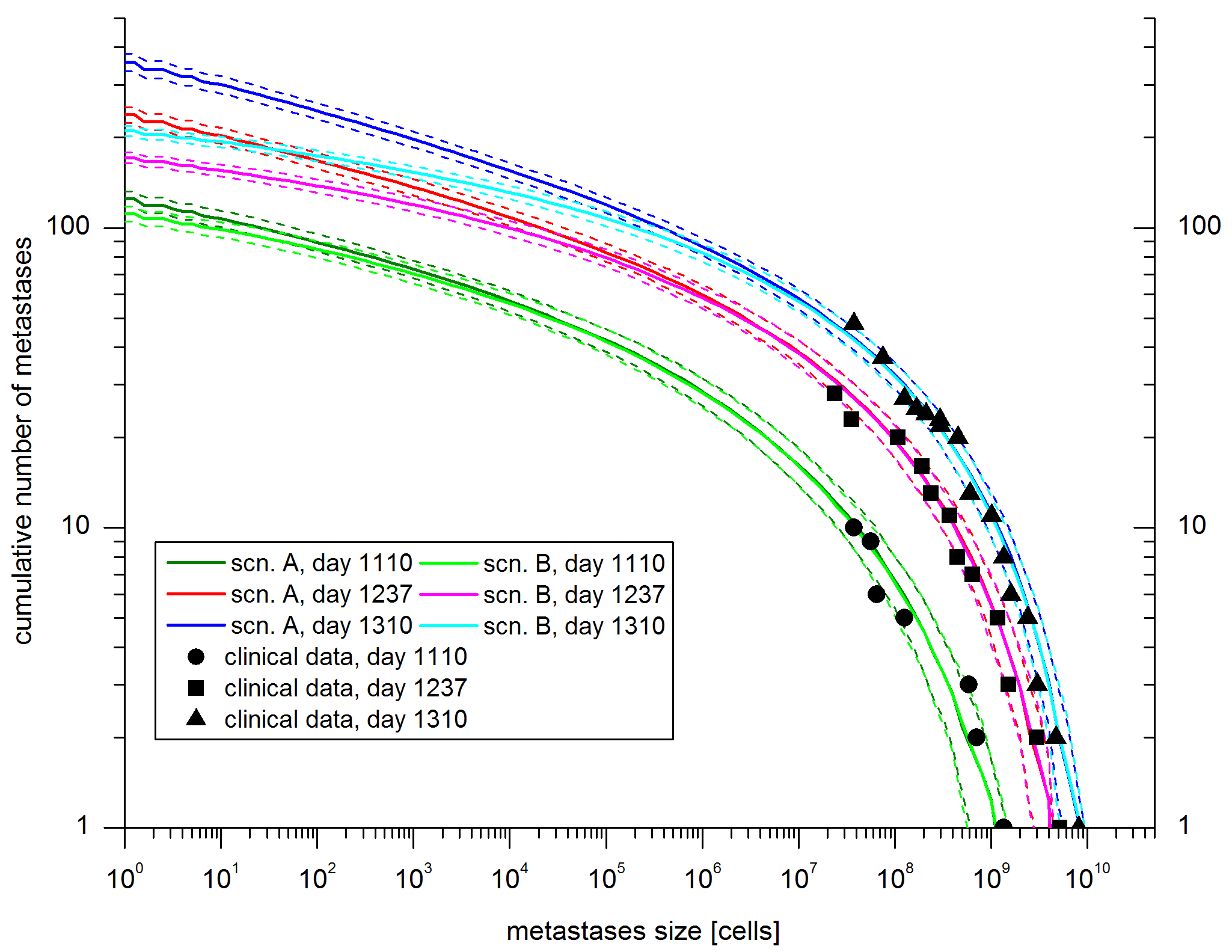

Supplement: Figure S3 — Comparison of scenario A and B. In this figure the simulation results of the scenarios A and B are displayed in the same graph. The thick lines represent the mean and the dashed lines the corresponding standard deviations. The graph clearly shows that in the range of the clinical data, the two scenarios A and B are nearly identical. Only in the range of the smaller metastases and late during the time course the scenarios can be separated. This is plausible, since tumours have to reach a certain minimal size, before they start metastasizing. Therefore, the effect whether metastases do metastasize can only be observed, after the first metastasis had grown large enough to start spreading metastases of its own. Including the standard deviation it is nearly impossible to clearly separate both scenarios at day 1110. At day 1237 both scenarios can be distinguished only for very small metastases from the size of 1 till 1000 cells. At day 1310 both scenarios can be clearly distinguished in the metastasis size range from 1 till 104 cells. If only the mean values are compared, the metastasis size ranges where both scenarios can be separated change as follows: day 1110→1–100 cells, day 1237→1–105 cells, day 1310→1–106 cells (see Fig. S3). (TIF) [file pone.0035689.s003.tif]

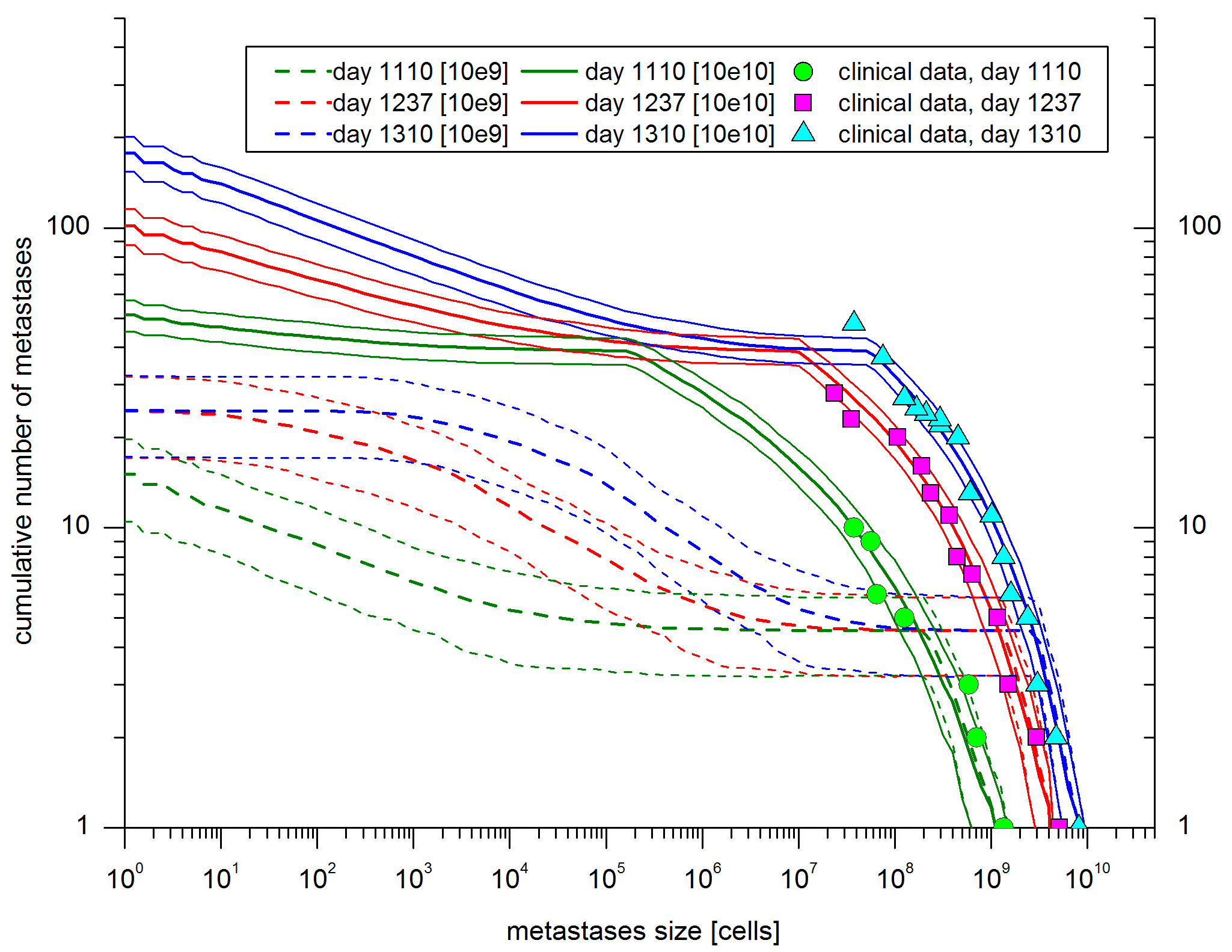

Supplement: Figure S4 — Simulation results of scenario C with mean and standard deviation. In scenario C both primary tumour and metastases are able to spread metastases. Cells that are disseminated from the primary tumour and from metastases after they reached a size of 109 cells (dashed lines) or 1010 cells (solid lines), respectively, lose their ability to form further metastases. The thick lines represent the mean values, while the thin lines above and beneath each thick line represent the corresponding standard deviation. The clinical data does not fit with the dashed lines, which indicates that cells that are disseminated from tumours larger than 109 cells are still able to form new metastases. In contrast, the clinical data fits well with the solid lines. This finding supports the assumption that cells that are disseminated from tumours larger than 109 cells may lose the ability to form metastases. However, the clinical data is not detailed enough to definitively decide this question. Clinical data from metastases smaller than 107 cells would be necessary to answer this question. The plateaus are caused by the primary tumour that reached the critical size of 109 or 1010 cells, resp. As long as the first metastases spread by the primary do not reach the minimal size to spread metastases of their own, no new metastases are created, which explains the plateau observed. After these metastases start spreading metastases themselves, the plateau dissolves and the number of metastases starts rising again. As soon as the first metastases reach the critical size, too, a second plateau starts to form which can be observed for the dashed lines (critical size = 109 cells). (TIF) [file pone.0035689.s004.tif]

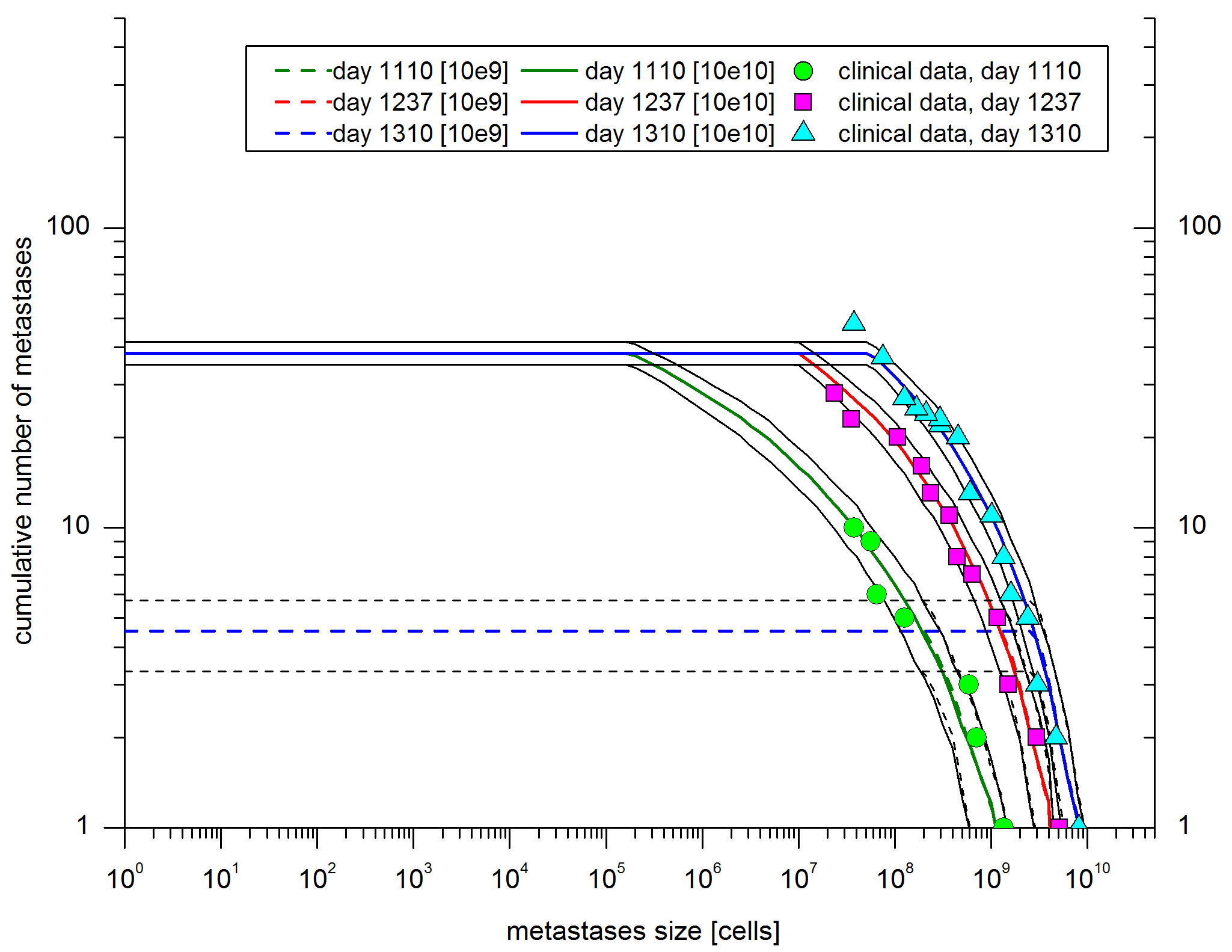

Supplement: Figure S5 — Simulation results of scenario D with mean and standard deviation. In scenario D the metastases are not able to metastasise. Similar to scenario C it is investigated whether cells that are disseminated from the primary tumour and metastases after they reach a size of 109 cells (dashed lines) or 1010 cells (solid lines), respectively, lose their ability to form metastases. The thick lines represent mean values, while thin black lines above and beneath each thick line represent the corresponding standard deviation. As in scenario C the clinical data does not fit with the dashed lines but with the solid lines. In contrast to scenario C the observed plateau remains, since only the primary tumour is able to metastasise. So, as soon as the primary tumour reaches the critical size of 109 or 1010 cell, resp, no new metastases are created. (TIF) [file pone.0035689.s005.tif]

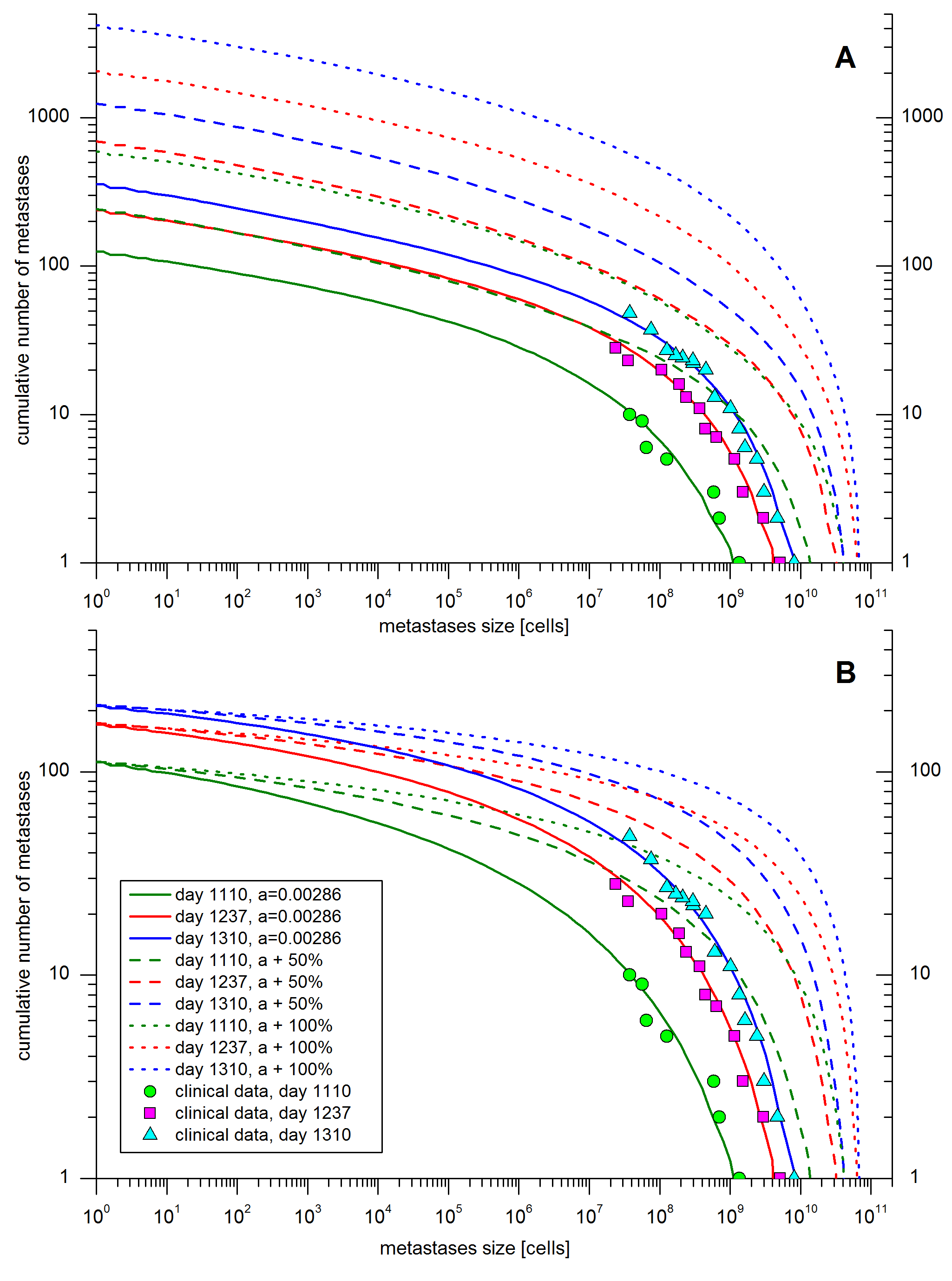

Supplement: Figure S6 — Simulation results of scenario A and B with higher growth rate for the metastases. The graphs show the simulation results for the scenarios A (metastases are able to metastasise) and B (metastases do not metastasise) with a varied growth rate for the metastases. The solid lines represent the simulation results for the case that primary tumour and metastases grow with the same growth rate of a = 0.00286 day−1. The dashed and dotted lines display the simulations results for the case that metastases grow with a 50% (a = a = 0.00429 day−1) or 100% (a = a = 0.00572 day−1), resp., higher growth rate than the primary tumour. The comparison of the simulation results with the clinical data clearly shows that in this case of a HCC metastases do not grow faster than the primary tumour, but that they in fact grow with the same growth rate as the primary tumour. (TIF) [file pone.0035689.s006.tif]

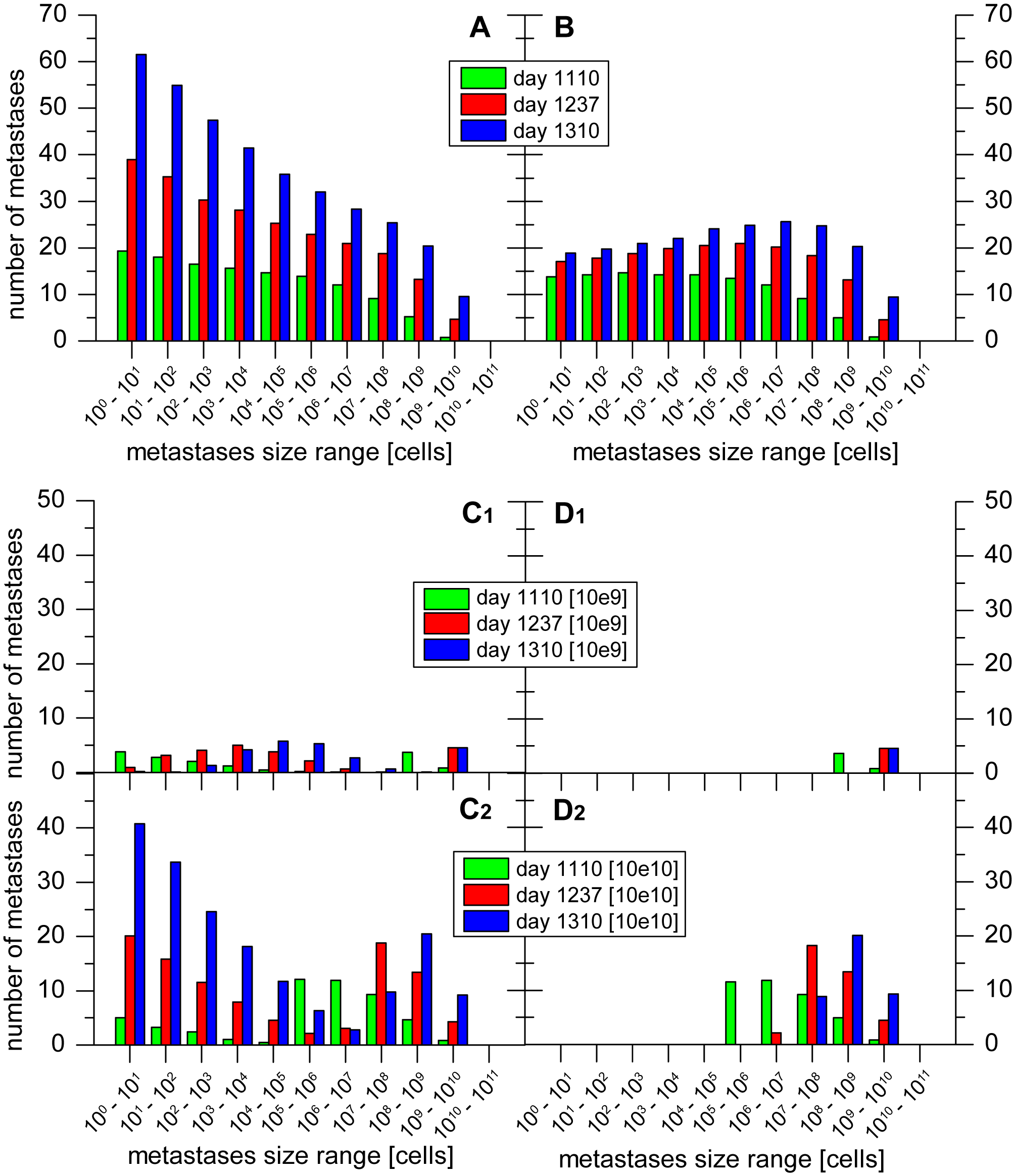

Supplement: Figure S7 — Frequency distribution of scenarios A–D. The graphs display the number of metastases belonging to the size ranges applied to the x axis. In scenario A much more new created metastases were present than in scenario B, indicating how much impact metastasising metastases gain on the number of metastases. In scenario B much fewer new metastases were created than in scenario A, since only the primary tumour was able to metastasise. The higher number of metastases for the bigger metastases sizes at the days 1237 and 1310 in scenario B results from the logarithmical division of the metastases size ranges. The graphs C1 and C2 clearly display the decrease of new created metastases after the primary tumour reached the critical size of 109 (C1) or 1010 (C2) cells, respectively, and how the number of new metastases slowly starts rising again after the first metastases started spreading metastases of their own. In graph C1 a second decrease of new created metastases can be observed for the days 1237 and 1310. In contrast to the first decrease, the second decrease occurs less sudden. This happens because at the time the first metastasis reached the critical size of 109 cells already multiple metastases are able to spread metastases. In contrast to scenario C only the primary tumour was able to spread metastases in scenario D. As a result no new metastases were created after the primary tumour reached the critical size of 109 (D1) or 1010 (D2) cells, respectively. This fact can be observed in the graphs D1 and D2. The existing metastases kept on growing, but no new metastases are created. The total number of metastases is the same for the three time points (4 in D1 and 38 in D2). (TIF) [file pone.0035689.s007.tif]
